# Targeting Circulating Tumor Cells in Pancreatic Ductal Adenocarcinoma: Rationale, Current Evidence, and a CEACAM6 CAR-T Strategy

**DOI:** 10.3390/cancers18111852

**Published:** 2026-06-05

**Authors:** Marcin Piejko, Karolina Bak, Joanna Wierciak, Hanna Plutecka, Natalia Wilczynska-Zawal, Malgorzata Osmola, Kamil Rapacz, Jacek Kijowski, Patrycja Mensah-Glanowska, Antoni Szczepanik, Marek Sierzega

**Affiliations:** 1 3rd Department of General Surgery, Jagiellonian University Medical College, 31-202 Krakow, Poland; antoni.szczepanik@uj.edu.pl; 2 Jagiellonian Center for Clinical Trial—Biobank, Jagiellonian University Medical College, 30-688 Krakow, Poland; karolina1.bak@uj.edu.pl (K.B.); joanna.wierciak@uj.edu.pl (J.W.); natalia.wilczynska@uj.edu.pl (N.W.-Z.); kamil.rapacz@uj.edu.pl (K.R.); 3 Division of Molecular Biology and Clinical Genetics, Jagiellonian University Medical College, 30-688 Krakow, Poland; hanka.plutecka@uj.edu.pl; 4 Maria Sklodowska-Curie Medical Academy in Warsaw, 03-411 Warsaw, Poland; mal.osmola@gmail.com; 5 General, Oncological, Gastroenterological and Transplant Surgery Clinical Department, University Hospital in Krakow, 30-688 Krakow, Poland; 6 Cell Bank, Malopolska Centre of Biotechnology, Jagiellonian University, 30-387 Krakow, Poland; jacek.kijowski@uj.edu.pl; 7 Department of Hematology, Jagiellonian University Collegium Medicum, University Hospital in Cracow, 30-688 Krakow, Poland; patrycja.mensah-glanowska@uj.edu.pl; 8 1st Department of Surgery, Faculty of Medicine, Jagiellonian University Medical College, 30-688 Krakow, Poland; marek.sierzega@uj.edu.pl

**Keywords:** PDAC, circulating tumor cells, CTCs, ctDNA, MRD, CEACAM6, CAR-T, adoptive immunotherapy

## Abstract

**Background**: Pancreatic ductal adenocarcinoma (PDAC) exhibits high post-resection relapse and early systemic dissemination rates. The level of circulating tumor cells (CTCs) correlates with early metastatic failure, motivating CTC interception strategies. **Methods**: In this hypothesis-driven review, we synthesized the contemporary evidence on PDAC staging and therapy, CTC detection (including portal versus peripheral sampling), and circulating tumor DNA (ctDNA)-based minimal residual disease (MRD), and evaluated the translational rationale for CTC-targeted adoptive immunotherapy focusing on CEACAM6 and CAR-T cells. **Results**: Prospective studies report higher portal versus peripheral CTC yields and stronger associations with relapse; tumor-informed ctDNA positivity in peri-operative and surveillance windows predicts shorter disease-free survival. CEACAM6 is overexpressed in PDAC and linked to invasion and metastasis, supporting antigen selection. However, target overexpression alone does not establish clinical suitability for adoptive cell transfer. Consequently, its therapeutic implementation must contend with assay heterogeneity, on-target/off-tumor risks, and the lack of interventional outcome data in PDAC, all of which remain key hurdles. **Conclusions**: CTC-targeting is biologically plausible and operationally measurable in PDAC. Consequently, a CEACAM6-directed CAR-T approach is proposed as a potential strategy for the interception of minimal residual disease (MRD). Randomized and biomarker-selected trials with composite MRD-clearance endpoints (CTC < LOQ and ctDNA-negative) may be justified to validate this interventional hypothesis.

## Simple Summary

Even when diagnosed early as a localized tumor, pancreatic ductal adenocarcinoma (PDAC) often spreads microscopically. This explains why many patients experience recurrence even after what appeared to be curative surgery. Cells that break away from the tumor and circulate in the blood—circulating tumor cells (CTCs)—can be detected in the blood. Because CTCs are viable and reflect tumor biology, therapies targeting them in operable patients may potentially reduce the risk of systemic relapse. In this hypothesis-driven review, we summarized current evidence on the staging and treatment of PDAC, explained what CTCs can tell us, and discussed whether targeting CTCs is a rational strategy. We outlined why CEACAM6 is a promising target and propose CEACAM6-directed CAR-T cells as a candidate therapy for minimal residual disease (MRD) in PDAC.

## 1. Introduction

Pancreatic ductal adenocarcinoma (PDAC) remains one of the most lethal solid tumors. In the United States, approximately 67,440 new cases and 51,980 deaths were projected for 2025, and pancreatic cancer is now the third leading cause of cancer-related death among all malignancies [1]. Its prognosis remains poor, with a 5-year relative survival of about 13% across all stages [2,3].

The poor prognosis of PDAC reflects both its clinical presentation and its underlying biology: the disease is frequently diagnosed at an advanced stage, often invades adjacent structures, particularly major vessels, and has a marked tendency for early dissemination (60% of patients are diagnosed with metastatic disease). Moreover, its aggressive behavior and desmoplastic, immunosuppressive tumor microenvironment have hindered durable responses to immunotherapy [1,4,5].

Among patients with resectable PDAC—precisely the cohort where cure is still conceivable—multiple prospective studies show that detection of circulating tumor cells (CTCs) correlates closely with early relapse, as well as shortened relapse-free survival and overall survival (RFS/OS) after surgery, indicating excellent prognostic value [6].

In the primary resectable cohort—where curative long-term outcomes are theoretically achievable—the systemic shedding of circulating tumor cells (CTCs) into both peripheral and portal vascular networks presents a formidable primary endpoint in peri-operative trials. While prospective tracking has established that subclinical baseline CTC levels are a highly sensitive prognostic indicator of early postsurgical relapse, this diagnostic value has historically failed to translate into actionable therapeutic interventions [7,8]. CTCs can colonize various body compartments and initiate metastatic spread despite macroscopically successful tumor resection and serve as a prognostic indicator of disease recurrence in PDAC patients. Excellent prognostication has not yet translated into therapeutic actionability; nevertheless, it underscores a rational scientific framework for evaluating whether early, biomarker-guided strategies targeting MRD can actively prevent systemic relapse [2,3].

To address this clinical problem, this review integrates a biological narrative that positions CTC not only as a prognostic factor but also as an therapeutic target. We map out a mechanistic continuum of vascular-phase metastasis in PDAC that links four distinct phases: (1) the early shedding of tumor cells into the portal vein, which establishes a disease both, on histological and cellular level; (2) the dynamic upregulation of the surface glycoprotein CEACAM6, which allows these suspended cells to evade anoikis (anchorage-independent cell death) during transit; (3) the subsequent homing and colonization of these surviving cells in the liver parenchyma; and (4) their unique vulnerability to intravascularly deployed CEACAM6-directed chimeric antigen receptor (CAR) T cells. By intercepting cells in suspension—where they are stripped of the protective, dense desmoplastic stroma and immunosuppressive microenvironment characteristic of solid PDAC masses—this strategy exploits the very biology of vascular dissemination to achieve total eradication of subclinical disease.

**Figure 1:**
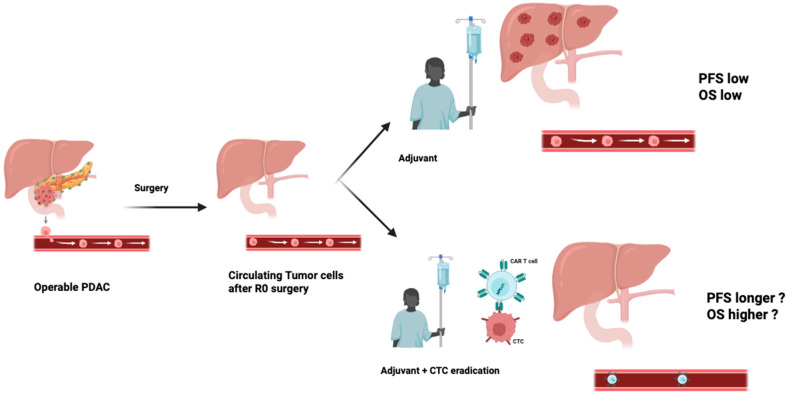
The rationality of CTC-targeted CAR-T therapy. Following surgical resection, residual CTCs frequently persist in the circulation, seeding occult micrometastases that drive disease recurrence and poor clinical outcomes despite adjuvant therapy. This subclinical survival manifests clinically as abbreviated progression-free survival (PFS) or disease-free survival (DFS) and compromised overall survival (OS). Intercepting this fluid phase through CTC-targeted eradication strategies, such as CAR-T cell-based immunotherapy, offers a rational framework that may enhance disease control and ultimately translate into prolonged PFS and OS. Figure 1 was created with BioRender.com.

Extensive reviews of adoptive cell therapies (ACT) in PDAC showcase a broad spectrum of emerging approaches, primarily centered on CAR-T cells. However, they also expose the profound physical and immunological bottlenecks that cripple cellular efficacy in solid tumors compared with hematologic malignancies, directly motivating a conceptual pivot toward an intravascular, CTC-first/MRD-first therapeutic interception strategy [9] (Figure 1).

In this review, we systematically searched PubMed/MEDLINE, Scopus, Web of Science, and ClinicalTrials.gov for peer-reviewed articles published through May 2026. Search strings incorporated combinations of the following Medical Subject Headings (MeSH) and keywords: ‘pancreatic ductal adenocarcinoma’, ‘circulating tumor cells’, ‘CEACAM6’, ‘chimeric antigen receptor T-cell’, and ‘minimal residual disease’. Literature was prioritized and evaluated based on its translational and mechanistic relevance. High priority was assigned to prospective clinical trials and pilot studies establishing the validity of CTC and ctDNA tracking in perioperative settings, and to preclinical data mapping cellular immunotherapies. Case reports, non-English publications, and studies without rigorous experimental validation or defined molecular controls were excluded. Through this selective framework, we synthesize the current state of PDAC staging, liquid biopsy, and CEACAM6 biology to establish a sound translational rationale for an intravascular CAR-T interception platform. This study aims to analyze whether targeting CTCs with CAR-T cells could be a rational approach to improving clinical outcomes in patients with PDAC.

Accordingly, the objective of this hypothesis-driven review is to synthesize contemporary clinical evidence of the prognostic roles of CTCs and ctDNA in MRD tracking. We evaluate how these biomarkers could potentially be leveraged to guide post-operative therapeutic interventions. Finally, we explore the translational feasibility of using a CEACAM6-targeted CAR-T cell platform as a candidate model to intercept circulating clones suspended in this fluid phase of MRD.

## 2. PDAC—Unmet Medical Need

### 2.1. Staging and Characteristics

The 5-year relative survival for pancreatic cancer across all stages is approximately 13%, the lowest among solid tumors [1]. However, survival varies significantly by stage: localized PDAC yields 44% 5-year survival, regional 17%, and distant ~3% [2,3].

PDAC, like other malignancies, is staged using the tumor-node-metastasis (TNM) system, currently the 8th edition, where the “T” category is defined by tumor diameter and its involvement of major arterial trunks (coeliac axis, superior mesenteric artery, and/or common hepatic artery). Regional lymph nodes are classified as N0 (no metastases), N1 (1–3 positive nodes), or N2 (4 or more nodes involved); while the M category denotes the presence or absence of distant metastases [10,11].

Clinical preoperative decision-making is based on resectability status, which defines tumors as resectable, borderline resectable, locally advanced/unresectable, or metastatic, with minor variation across staging systems [12,13,14,15,16,17]. Local tumor extension is defined by major vascular infiltration and the feasibility of resection and reconstruction. Current therapeutic algorithms are outlined in the NCCN Guidelines v2.2025 [18,19].

Only about 20% of PDAC patients present with a primary resectable tumor amenable to margin-negative (R0) surgery [20], typically combined with adjuvant systemic therapy [15].

Borderline resectable tumors feature limited vascular involvement (e.g., abutment or short-segment encasement of venous structures with potential for reconstruction, or arterial contact below thresholds of unresectability), where an upfront R0 resection is uncertain without vascular resection [21,22]. In these cases, induction therapy (systemic treatment ± radiation) is used to select for favorable biology, increase the R0 rates, and spare futile surgery [13,21,22].

Locally advanced disease involves extensive vascular infiltration precluding primary reconstruction, where systemic downstaging may later allow surgery [23]. Patients with metastatic disease involving either distant organs (mostly liver and lungs) or non-regional lymph nodes (TNM Stage IV) are similarly unresectable [24].

About 20% of PDAC are primarily resectable; furthermore, around 20% of patients with unresectable non-metastatic PDAC might undergo resection following induction therapy [25]. However, recurrence rates remain high even after curative resections, and previous attempts to develop clinically relevant prognostic predictors have yielded inconclusive results. This indicates the need to develop other modern monitoring and prognostic biomarkers. The CLUSTER study shows that high preoperative peripheral blood CTC counts strongly correlate with early recurrence, establishing CTCs as a promising biomarker of disease relapse (Table 1) [6]. It also highlights the imperative for MRD-targeted interventions to intercept disease recurrence after surgery [19].

### 2.2. Current Standard of Care in the Management of PDAC–Systemic Treatment

#### 2.2.1. Adjuvant

For fit patients after R0/R1 resection, the PRODIGE trial demonstrated a survival benefit with an adjuvant therapy with mFOLFIRINOX (modified leucovorin–5-fluorouracil–irinotecan–oxaliplatin), with median OS 53.5–54.4 months versus ~35 months with gemcitabine and consistent gains in PFS and metastasis-free survival, and is now the standard of care [26,27]. For patients ineligible for mFOLFIRINOX, results from an ESPAC-4 trial confirm GemCap (gemcitabine + capecitabine) superiority over Gem (median OS 31.6 versus 28.4 months), with pronounced benefit in R0 and node-negative subgroups [28]. Frail patients should receive gemcitabine monotherapy as an adjuvant treatment [23].

Currently, an ongoing phase II APOLLO trial evaluates olaparib (a poly[ADP-ribose] polymerase inhibitor, PARPi) versus placebo as adjuvant treatment in patients with resected PDAC harboring pathogenic *BRCA1*, *BRCA2*, or *PALB2* mutations, but the final results from this study have not yet been published [29].

#### 2.2.2. Induction Therapy

Preoperative treatment for patients with pancreatic cancer can be divided into induction therapy for borderline or locally advanced PDAC, which aims to downsize the tumor, while neoadjuvant treatment can be proposed to patients where upfront surgery is possible in clearly resectable disease [23]. In borderline resectable PDAC, neoadjuvant therapy (commonly mFOLFIRINOX or Gem-based ± radiotherapy) improves R0 rates and survival versus upfront surgery. In resectable PDAC, the benefit from chemotherapy is debated, and patient selection (tumor size, CA19-9, nodal status) is critical [30,31]. In the event of borderline resectable PDAC, there is evidence supporting the use of induction treatment over upfront surgery [32]. Induction therapy may also help downsize the tumor in locally advanced PDAC, facilitating resection; a meta-analysis reported a pooled resection rate of ~26% [33]. The phase II JCOG1407 trial comparing mFOLFIRINOX vs. Gem plus nab-paclitaxel in locally advanced PDAC showed a modest superiority of the latter in this stage [34].

#### 2.2.3. Systemic Treatment in Metastatic Disease

For metastatic/locally advanced disease, first-line palliative FOLFIRINOX, NALIRIFOX (liposomal irinotecan–5-FU–LV–oxaliplatin), or Gem + nab-paclitaxel remains the reference backbone [35,36,37]. Treatment choice should take into account the patient’s performance status, comorbidities, laboratory test results (notably bilirubin level), and potential toxicity [18,38]. In locally advanced PDAC, electrotherapy (e.g., Tumor Treating Fields [TTFields], Optune Pax^®^, Novocure GmbH, Baar, Switzerland) combined with Gem and nab-paclitaxel modestly improved OS (FDA approval in 2026) [39].

#### 2.2.4. Targeted Therapy: Small Molecules

At present, the role of precision medicine in PDAC remains limited, as only a minority of patients are eligible for and benefit from such therapies, albeit sometimes with durable responses. In this context, RAS inhibitors are among the most promising emerging strategies. Recognized biomarkers and molecular targets are as follows: patients with germline *BRCA1/2* mutations may respond to maintenance treatment with PARPi (olaparib) [40]. Similarly, another PARPi, rucaparib, showed efficacy in patients with BRCA1/2 or PALB2 somatic or germline mutations [41].

Tumors with high microsatellite instability/mismatch repair deficient (MSI-H/dMMR) PDAC may respond to immunotherapy with pembrolizumab [42]; tumors with *NTRK* fusions may benefit from TRK inhibitors (e.g., larotrectinib, entrectinib, repotrectinib) [43].

Since activating *RAS* mutation is present in approximately 90% of PDACs [44], targeting this pathway is particularly tempting. Recent data from a Phase I/II trial evaluating the RAS(ON) multi-selective inhibitor daraxonrasib demonstrated encouraging clinical activity, yielding a median PFS of 8.5 months and a median OS of 13.1 months in previously treated patients with metastatic PDAC, albeit with a non-negligible toxicity profile [45]. *KRAS* p.G12C (present in 1–2% of PDAC) targeting with sotorasib, showed median PFS of 4 months and OS of 6.9 months in a phase I/II trial [46]. *KRAS* G12D inhibitors (e.g., MRTX1133) demonstrated strong preclinical synergy with checkpoint blockade, but the first-in-human program was terminated early for formulation issues, underscoring clinical hurdles and the need for rational combinations (e.g., SHP2/PI3K co-inhibition) [41,42,43]. Another interesting target in PDAC is the c-MET protein. Recently presented early results of an antibody–drug conjugate (ADC) telisotuzumab adizutecan showed encouraging antitumor activity in a phase I study [47].

#### 2.2.5. Targeted Therapy: Antigen-Directed Cellular and Antibody-Based Therapies

PDAC shares several tumor-associated antigens (TAAs) with other gastrointestinal malignancies (Table 2), three of which are particularly noteworthy. CEACAM6 is a highly prevalent antigen, expressed in approximately 90% of PDAC tumors [48,49]. Nevertheless, the expression of CEACAM6 on macrophages, particularly pulmonary macrophages, can lead to pulmonary and hematological toxicity (on-target/off-tumor toxicity). The variability in CEACAM6 (and other antigen) expression highlights the importance of targeted antigen expression in each patient undergoing antigen-directed therapy [48,50]. Furthermore, TROP2 and Nectin-4, which are present in roughly 50% of PDAC cases, are versatile targets for both CAR-T cells and established ADCs. Examples include the TROP2-directed sacituzumab govitecan, currently approved for breast and urothelial cancers, and the Nectin-4-directed enfortumab vedotin, primarily indicated for urothelial cancer [51].

#### 2.2.6. Targeted Therapy: Adoptive Immunotherapy

Unlike hematologic malignancies, where CAR-T cells are widely adopted, PDAC’s desmoplastic stroma, immune exclusion, and antigen heterogeneity blunt the intratumoral delivery and persistence of cell therapies. CAR-T consists of two main elements: lymphocytes (autologous or allogeneic) and a chimeric antigen receptor (CAR). CAR is an artificial construct engineered with an antigen-recognizing element of an antibody and a downstream signaling domain that transduces a signal to the lymphocyte’s cytoplasm. Activating CD3 on lymphocytes mimics TCR-based activation, resulting in a cytolytic response or the recruitment of other effector cells, such as macrophages. 

Contemporary reviews catalog early-phase efforts in CAR-T, CAR-NK, TCR-T, and TILs, but routine durable responses are not yet established [9,62]. Off-the-shelf CAR invariant natural killer T (iNKT) cells show potent, graft-versus-host disease (GvHD)-sparing activity in preclinical PDAC models and are highlighted as translationally promising [63]. Oncolytic adeno-immunotherapy with human epidermal growth factor receptor 2 (HER2) CAR-NK enhances NK persistence and tumor control in PDAC xenografts, exemplifying the trend to combinatorial immuno-engineering [64].

### 2.3. Follow-Up and Markers of Early Relapse in PDAC: CTCs and Beyond

Patients after PDAC resection have a high risk of relapse after surgery (~80%). For patients with resected PDAC, ESMO guidelines recommend regular follow-up, as patients in active surveillance programs are more likely to have recurrence detected at an asymptomatic stage [65]. Evidence regarding the impact of this approach on OS remains insufficient. There is no proposed surveillance schedule nor imagery technique. In oncological practice, computed tomography (CT) is routinely used alongside assessment of the carbohydrate antigen (Ca 19.9) marker. Outside of that, currently, oncologists have no other tools to assess early relapse risk. In this context, assessment of the MRD may come in handy.

#### 2.3.1. Minimal Residual Disease in PDAC

In a clinical and translational context, MRD in solid oncology represents the persistence of subclinical tumor burden following curative-intent interventions, such as surgical resection, neo/adjuvant chemoradiation. The term MRD is highly adapted in hematology, where it serves as a biomarker for treatment intensification. MRD identifies residual malignant elements that are below the detection threshold of conventional cross-sectional imaging (CT or Positron Emission Tomography). MRD relies on “liquid biopsies” that sample various tumor-derived materials from the blood: including ctDNA (Circulating Tumor DNA), CTCs, and others (like exosomes and disseminated tumor cells, DTCs).

##### Example: Learning from Hematology: Blinatumomab

In MRD-positive B-cell precursor acute lymphoblastic leukemia B-ALL in hematologic remission, blinatumomab (immunotherapy from the bispecific T-cell engager group) achieves ~78% MRD clearance with associated improvements in relapse-free and overall survival, and underpinned regulatory acceptance of MRD as an actionable disease state [66,67]. Subsequent experiences—including low-level MRD detected by next-generation sequencing (NGS) and combinations-reinforce the notion that eradicating MRD can alter the natural history [68,69]. The principle is transferable: treat when the disease is minimal, measurable. In PDAC, portal CTCs and ctDNA-MRD define the measurable states [7,70]. While profound biological and microenvironmental differences distinguish hematological malignancies from solid tumors like PDAC, certain conceptual MRD management strategies can be adapted from one domain to another.

#### 2.3.2. CTCs

Serial monitoring of patients facilitates the identification of MRD-positive cohorts and provides a more comprehensive understanding of the clinical course of PDAC. A clear clinical rationale exists for the development of CTC-targeted therapies. CTCs constitute a heterogeneous cell population reflecting the vast biological diversity of PDAC, including subpopulations with low proliferative potential that may evade conventional chemotherapy. Consequently, effective CTC eradication requires highly potent cytolytic effector mechanisms, such as Chimeric Antigen Receptor (CAR) T-cell therapy targeting specific CTC surface antigens.

The eradication of CTCs during the postoperative period represents a critical therapeutic window to reduce the risk of early relapse in patients following R0 resection. However, this clinical objective requires CTC-targeted therapies that enable rapid recognition and cytolytic elimination (“catching and killing”) of disseminated cells. The goal is to effectively intercept them before they colonize distant metastatic niches [7,71]. Such a strategy is fundamental to address the so-called ‘vascular phase’ of PDAC progression and improve long-term outcomes in primarily resectable disease.”

In resectable (TNM I/II) cohorts where cure is still plausible, portal venous blood (PoVB) CTC positivity tightens the link to early relapse and adverse RFS/OS, supporting CTC-guided risk stratification peri-operative intensification, and use of CTC-based MRD endpoints (e.g., CTC < LOQ at prespecified windows) [7,72]. Also, other studies, such as the already mentioned CLUSTER trial, provide evidence that CTC level correlates with risk of early relapse. In the resectable PDAC group, patients who relapse up to 12 months post-surgery present with >7 epithelial CTCs or >1 mesenchymal-type CTC/mL of peripheral blood (PB) [6] (Table 1). Consequently, incorporating standardized CTC assessment protocols into post-operative surveillance frameworks could be investigated as a potential strategy to better risk-stratify patients at risk of early relapse. In a typical scheme, sampling of PB and PoVB before the surgery at D-7/D-1 as well as D+14 and monthly up to 2 years, will help distinguish two groups of patients—with MRD-positive from MRD-negative ones.

#### 2.3.3. ctDNA

Another biomarker with prognostic and predictive capabilities in patients with PDAC is ctDNA [73]. Positivity of ctDNA in the peri-operative MRD window and during surveillance predicts markedly shorter disease-free survival (DFS), often preceding radiologic and clinical recurrence by months. Its role has been explored in both resectable and unresectable PDAC, and is already being integrated into clinical trials, although it is not yet widely used in routine practice. A key feasibility question is which ctDNA alterations to select for assessment, in part because ctDNA alteration burden is higher in advanced than in resectable disease [74]. ctDNA has some advantages over tissue DNA analysis, including potential real-time monitoring of treatment response. On the other hand, the small amount of tumor DNA in circulation also limits detection. The most commonly altered genes in PDAC are TP53, KRAS (~50% of each), CDKN2A, and GNAS (<10%, each) [74]. Pathogenic variants at diagnosis were detected in patients with a poorer outcome and correlated with response to neoadjuvant therapy in borderline PDAC [75].

In summary, CTC and ctDNA monitoring can play prognostic and predictive roles in patients with PDAC and help guide clinical management [70,76,77].

## 3. Discussion

Two converging observations motivate a CTC-targeted approach in PDAC. First, PDAC behaves as a systemic disease early in its course; even when resection is feasible, distant relapse is common, consistent with occult dissemination at (or before) diagnosis [78]. Second, CTCs—especially PoVB—carry strong prognostic information and align with subsequent distant failure after surgery [7,72]. If CTCs mark the biology that kills, selectively removing or neutralizing them (and related MRD) could plausibly lower relapse risk in feasible patients. Nevertheless, it must be emphasized that in advanced-stage patients, CTCs burden typically reflects the metastatic process that has already established radiologically overt or occult secondary lesions. In late-stage lesions, therapeutic clearance of CTCs may offer negligible utility. Instead, the therapeutic window for active CTCs interception is likely confined to early-stage, resectable disease. In the perioperative setting, combining macroscopic surgical resection with intravascular cellular interception creates an opportunity to achieve the complete systemic eradication of residual PDAC cells before metastatic colonization.

### 3.1. Clinical Rationale of CTC Targeting

Targeting CTC in operable PDAC patients because of the correlation between CTC level after surgery and risk of early relapse seems plausible [7,72]. Additionally, CTC enumeration and phenotyping before surgery and at follow-up provide sensitive tools for detecting relapse [79,80]. Complementarity with ctDNA detection in the peri-operative window and during surveillance correlates with markedly shorter RFS and precedes imaging; combining CTC and ctDNA readouts can improve sensitivity/specificity for residual disease and guide intensification [70,76,77].

### 3.2. The Mechanistic Continuum of Liquid-Phase Metastasis and CAR-T Interception

The transition from localized PDAC to metastatic recurrence is a coordinated biological cascade in which portal venous shedding, biomarker-based MRD, CEACAM6 signaling, and CAR-T surveillance converge mechanistically.

Dissemination: Early in pathogenesis, epithelial-to-mesenchymal transition (EMT) drives the shedding of malignant cells into the portal vein, establishing a subclinical MRD state that is tracked systemically via peripheral ctDNA. Once suspended in the high-shear environment of the circulation, these cells face anoikis—programmed cell death triggered by the loss of matrix anchorage. To survive, PDAC CTCs upregulate the surface glycoprotein CEACAM6 [48,49,50,81]. Homophilic clustering of CEACAM6 within membrane lipid rafts coordinates the recruitment of caveolin-1, hyperactivating the c-Src/Focal Adhesion Kinase (FAK) axis. This proxy signaling stimulates downstream PI3K/Akt cascades, suppressing mitochondrial cytochrome c release and caspase-3 activation. This molecular shield confers robust anoikis resistance on floating CTCs during transit.

Hepatic Seeding and Cellular Interception: These surviving, anoikis-resistant portal CTCs drain directly into the liver microvasculature to initiate metastatic colonization. However, this transit phase represents a profound therapeutic window of vulnerability. Unlike solid PDAC lesions, which construct physical and immunological barriers via dense desmoplastic stroma and immunosuppressive myeloid cells, the liquid phase of MRD lacks these defenses. In the bloodstream, the CEACAM6 antigen is fully exposed and globally accessible. Intravascularly deployed CEACAM6-targeted CAR-T cells bypass tissue-trafficking bottlenecks, rapidly forming immunological synapses with suspended CTCs to execute unhindered perforin- and granzyme-mediated lysis, effectively breaking the metastatic chain before colonization can solidify.

### 3.3. Causal Metastatic Vehicles vs. Passive Surrogate Biomarkers

To validate the therapeutic rationale of a CTC-targeted cell therapy, a critical distinction must be made between CTCs as passive surrogate indicators of aggressive tumor biology versus active, causal mediators of metastasis.

The intravascular tumor cell pool is profoundly heterogeneous. Mass tissue shedding by aggressive primary tumors releases millions of epithelial cells into the bloodstream. However, pioneering work in cancer biology has demonstrated that hematogenous metastasis is an extraordinarily inefficient process, with fewer than 0.01% of disseminated cells successfully establishing secondary lesions [82,83]. Most individual CTCs cannot withstand hydrodynamic shear stress or loss of extracellular anchorage and rapidly undergo apoptotic degradation [83]. Consequently, this bulk, short-lived population represents a passive surrogate biomarker—an immunological and physical echo of primary tumor burden rather than an active metastatic threat.

Conversely, hematogenous metastasis relies entirely on a minute, highly specialized subset of CTCs possessing stem-like properties and distinct survival mechanisms, termed metastasis-initiating cells [84,85]. This subpopulation acts as the obligate, causal mediator of distant organ colonization. Within this frame of reference, our proposed strategy shifts the utility of CTCs from a passive prognostic readout to an active therapeutic target. By targeting CEACAM6, a surface glycoprotein that dynamically drives anchorage-independent survival and liver-directed homing in pancreatic adenocarcinoma [86], the CAR-T avoids targeting benign cellular debris or passive surrogates. Instead, it may act as a selective filter to intercept the specific, functional drivers of metastasis during their vulnerable transit phase, thereby breaking the causal chain of systemic recurrence.

### 3.4. CTC Monitoring Challenges

While CTC monitoring offers many advantages, some issues should be addressed in this field. CTC detection remains platform-dependent. EpCAM-only capture can miss EMT-shifted cells and clusters, and absolute thresholds vary by method and compartment—complicating trial eligibility and response criteria [71]. Also, patient sampling and logistics should be carefully managed. Portal sampling requires endoscopic ultrasound (EUS) expertise and carries procedural risk (albeit low in pilot series), which may limit broad dissemination or serial testing outside specialized centers [80]. On the other hand, PB sampling has lower sensitivity in CTC quantification [7]. Cell-based detection methodologies, such as flow cytometry or size-based filtration, require relatively rapid sample delivery to the lab. Assay standardization is also crucial for CTC with longitudinal sampling to overcome reproducibility and sensitivity limits of single landmarks, particularly given the change in CTC phenotype from mostly epithelial (EpCAM-positive) to mesenchymal (vimentin-positive) [71,77]. Even when CTCs are accessible, detection of primary tumor or micrometastasis in PDAC is hindered by stromal exclusion, antigen heterogeneity, and immunosuppression [9]. Finally, there is a clinical-outcome evidence gap. Prognostic links are strong, but interventional data showing that targeting CTC improves OS in PDAC are not yet available.

### 3.5. CEACAM6 as a Target and CAR-T as the Effector

CTC-targeting needs an optimal effector. CAR-T has rational arguments, including the elimination of CTCs independent of proliferative potential and the elimination of these cells immediately after antigen recognition. Choosing an antigen specific to CTCs is a compromise between specificity, high abundance in the tumor, and its prevalence among PDAC patients. CEACAM6 is consistently upregulated in PDAC; it drives invasion, anoikic resistance, and metastatic competence through SRC/FAK/PI3K-AKT and ERK/MAPK programs. Genetic silencing reduces invasiveness and tumor growth in models [48,49,86]. Multi-omics analyses suggest overexpression in both basal and classical PDAC, with adverse prognosis, supporting broad relevance [52]. Preclinical approaches (e.g., siRNA/miR-29a via pHLIP) demonstrate suppression of tumor growth, highlighting the potential of targeted CEACAM6 as a drug target [50].

Nevertheless, CEACAM6 is present on normal epithelia and myeloid cells. It is enriched in some gastrointestinal mucosa, raising on-target/off-tumor concerns if high-affinity targeting is used. Cross-reactivity within the CEACAM family adds complexity [87,88]. Risk-mitigation options include affinity-tuned binders, logic-gated dual CARs, regional delivery (e.g., intratumoral), transient expression (mRNA CAR-T), and suicide switches [9].

### 3.6. Comparative Analysis of Alternative CTC-Neutralizing Modalities

While this proposal centers on a CEACAM6-targeted CAR-T strategy, several alternative therapeutic modalities have been explored or proposed for minimal residual disease clearance and CTC neutralization [9,64]. These include bispecific T-cell engagers (BiTEs) [9], ADCs [47], combinational oncolytic virotherapy with natural killer (NK) cells [64], and extracorporeal apheresis filtration systems [89].

#### 3.6.1. Bispecific T-Cell Engagers (BiTEs) and Soluble T-Cell Engagers (TCEs)

Bispecific constructs designed to cross-link CD3 on endogenous T cells with tumor antigens (such as emerging anti-CEACAM5/6 biAbs) represent a highly potent off-the-shelf modality [9]. However, soluble BiTEs possess a short serum half-life, necessitating complex, continuous intravenous infusion protocols. More critically, BiTEs lack autonomous, target-dependent self-regulation. To effectively neutralize ultra-rare CTCs scattered throughout the blood volume, high and sustained systemic concentrations of BiTEs are required [71]. This uncoupling of drug concentration from actual tumor burden drastically amplifies the risk of “on-target, off-tumor” systemic toxicity in healthy tissues that express low baseline levels of CEACAM6, such as normal pulmonary or gastrointestinal epithelia.

#### 3.6.2. Antibody-Drug Conjugates (ADCs)

ADCs (such as the anti-CEA/CEACAM6 conjugate EBC-129) utilize monoclonal antibodies to deliver highly potent cytotoxic payloads (e.g., monomethyl auristatin E) directly to malignant cells [47,51]. While highly effective against debulked or localized masses, ADCs are pharmacokinetically disadvantaged against solitary, floating CTCs. Because CTCs typically occur at extraordinarily low densities (1–10 cells per mL of whole blood), the likelihood of a fixed-dose, decaying ADC molecule establishing successful stochastic contact and subsequent receptor-mediated internalization in suspension is low [71]. Furthermore, the payload delivery mechanism does not result in local immune amplification, and systemic toxicities (e.g., bone marrow suppression or hepatotoxicity) make ADCs less suitable for long-term proactive surveillance in the fragile post-operative adjuvant window [47].

#### 3.6.3. Oncolytic Viruses (OVs) and CAR-NK

Integrating oncolytic virotherapy with cellular components, such as CAR-NK cells, has emerged as a strategy to turn immunologically “cold” solid tumors “hot” by leveraging direct viral lysis to recruit effector cells [64]. However, this synergy is biologically contingent upon a dense, localized solid tissue architecture [9,64]. Oncolytic viruses require high-density, cell-to-cell infection vectors to replicate, propagate, and shed secondary virions [64]. Isolated CTCs or minute microemboli suspended in a high-shear bloodstream cannot support this viral amplification loop [71,83]. Additionally, systemic delivery of OVs into the blood results in rapid neutralization by circulating complement factors and anti-viral antibodies, rendering them highly inefficient at targeting the liquid phase of MRD [9,71].

#### 3.6.4. Apheresis-Based Extracorporeal CTC Removal Devices

Experimental mechanical interception strategies utilize modified apheresis or leukapheresis units integrated with microfluidic chips, size-based nano-microsieves, or immunomagnetic filtration to physically remove CTCs from patient blood [71,89]. While appealing due to the total absence of biochemical systemic toxicity, apheresis is strictly constrained by a narrow temporal window [89]. It can only filter the blood volume passing through the circuit during the live, 2 to 4 h procedure [89]. Because CTC shedding from occult micro-metastatic deposits is highly intermittent and unpredictable, apheresis devices remain entirely blind to cells shed after the session ends [71,83]. They are also incapable of neutralizing CTC clusters that have already extravasated or lodged within the intricate capillary beds of the portal vein or hepatic parenchyma [7,83,85].

### 3.7. The Definitive Advantage of CAR-T Cells as Living Intravascular Patrols

CAR-T cell therapy fundamentally overcomes the limitations of these transient, passive, or localized systems by operating as an active, self-regulating biological system [9,90]. Because T cells naturally possess active extravasation, rolling, and homing capabilities within the vasculature, they are natively optimized to patrol the liquid phase [9,71]. Crucially, CAR-T cells exhibit conditional clonal expansion: when a CAR-T cell encounters a rare CEACAM6+ CTC, it triggers a localized proliferative cascade, amplifying the local therapeutic force precisely where and when the target is present [48,90]. Finally, the differentiation of a fraction of these cells into central and effector memory subsets ensures durable, ongoing immunosurveillance across months [9,62]. This active persistence allows the cellular graft to neutralize sporadic, delayed waves of CTC shedding throughout the critical post-surgical recurrence window, providing a curative potential unmatched by any non-living therapeutic platform [7,71,83].

### 3.8. Evidence Stratification: Clinically Validated Benchmarks vs. Proposed Hypotheses

To preserve translational objectivity, a strict distinction must be maintained between clinically established data, preclinically verified mechanisms, and the forward-looking therapeutic proposals advanced in this manuscript. The components underpinning our framework exist across distinct tiers of biological and clinical validation.

Clinically verified milestones include the systemic value of post-operative ctDNA-MRD detection for predicting rapid metastatic recurrence [70], alongside the technical feasibility and safety of EUS-guided portal venous sampling to isolate dense frontline CTC cohorts [79]. Similarly, the consistent overexpression of CEACAM6 in resected human PDAC specimens and its direct correlation with lymph node metastasis and abbreviated survival represents an established clinical reality [91]. Sitting beneath these are preclinically validated mechanisms, heavily documented in vitro and in vivo, which prove that surface clustering of CEACAM6 dynamically drives c-Src/FAK proxy survival signaling to enforce robust anoikis resistance during detachment [86].

Conversely, the final layer represents our speculative therapeutic proposal: the deployment of a CEACAM6-targeted CAR-T cell graft as an intravascular “liquid filter” to eliminate subclinical MRD before hepatic seeding. While early-phase clinical data confirm that CAR-T cell therapies can successfully engage surface targets in solid malignancies, their specialized execution within high-shear vascular streams to arrest minimal residual disease remains a conceptual model [9,90,92]. Acknowledging this evidence gradient is critical for guiding future trial designs and translating this roadmap into human applications.

### 3.9. Current Limitations and Barriers to Field Harmonization

Translating an intravascular, CTC-targeted CAR-T cell proposal into clinical reality requires a candid appraisal of the severe methodological, clinical, and biological limitations that currently fragment the fields of liquid biopsy and adoptive cell immunotherapy.

#### 3.9.1. Methodological Heterogeneity in CTC Enrichment and Detection

A paramount bottleneck preventing the clinical adoption of CTC-guided strategies is the extreme lack of standardization across isolation technologies. Published literature in PDAC reports baseline CTC detection rates ranging from a dismal 11% to a highly sensitive 92% [93]. This massive variance is driven by a fundamental split in enrichment principles. Traditional label-dependent platforms, such as the Food and Drug Administration (FDA)—approved CellSearch system, rely on positive immunomagnetic selection targeting the Epithelial Cell Adhesion Molecule (EpCAM) [93]. However, during hematogenous dissemination, aggressive PDAC cells routinely activate EMT, shedding their epithelial traits and drastically downregulating EpCAM. Consequently, antibody-based capture assays suffer from a high false-negative rate, systematically missing the most metastatic clones. While physical selection systems (isolating cells via size-exclusion membranes, microfluidic chips, or electric charge) circumvent marker dependency, they are frequently contaminated by background leukocytes. This profound technological heterogeneity complicates cross-study comparisons and underscores the critical need to transition to functional, targetable surface markers such as CEACAM6 that remain actively expressed during the mesenchymal transit phase.

#### 3.9.2. The Interventional Evidence Void in Liquid Biopsy

Although dozens of prospective observational studies and clinical registries have established that both peripheral and portal venous CTC burden serve as a powerful prognostic biomarker for disease recurrence, the field remains entirely devoid of interventional validation. Clinical oncology has yet to prove that actively treating or clearing a patient’s CTC burden translates into prolonged PFS or OS. While early-phase device feasibility protocols have emerged—such as the 2025/2026 OSCAR I clinical trial (NCT06481397) evaluating mechanical extracorporeal blood purification to filter cells in refractory cohorts [89]—there are no completed trials using targeted cellular or molecular therapeutics to actively intercept liquid-phase MRD. Our perspective represents a conceptual pivot designed to break this deadlock, migrating liquid biopsy from a passive, sensory risk-stratification tool into an actionable, biomarker-guided therapeutic target.

#### 3.9.3. Convergence with Classic Solid-Tumor CAR-T Barriers

Finally, the proposed platform must contend with the realities of cellular therapy kinetics. While targeting cells in suspension eliminates the physical barriers posed by dense desmoplastic tissue, it introduces a strict temporal dependence. If circulating CEACAM6+ CTCs extravasate rapidly and nest within the hepatic or peritoneal parenchyma before encountering the cellular graft, the rules of solid-tumor engagement are immediately reinstated. Once tucked inside a solid metastatic niche, the CAR-T cells will immediately face the classic bottlenecks that have historically blunted ACT in PDAC: severe antigen heterogeneity leading to target-negative escape variants, continuous antigen exposure causing tonic T-cell exhaustion, and a highly hostile tumor microenvironment dominated by regulatory T cells and myeloid-derived suppressor cells (MDSCs) [90,92]. Therefore, the therapeutic success of this strategy is heavily contingent upon precise clinical timing, requiring deployment strictly during the immediate post-operative window when the cancer is confined to its most vulnerable, vascular phase.

### 3.10. What Would a Rational Trial Look Like?

Designing a clinically sound trial roadmap requires careful calibration of patient selection criteria, manufacturing kinetics, and standardized monitoring endpoints. Therapeutic interception should target patients who present with a primary resectable or borderline-resectable tumor architecture but exhibit an exceptionally high risk of early systemic recurrence due to elevated perioperative CTC burden [7,70,72].

The proposed therapeutic sequence integrates curative-intent surgical resection with early perioperative CAR-T cell infusion (Figure 1). While cellular clearance is hypothesized to extend RFS and OS, rapid post-resection administration is vital. Combining immediate fluid-phase filtration with surgical debulking maximizes the probability of clearing residual disease at the single-cell level, protecting patients before microscopic niches can establish irreversible tissue colonization. To overcome the rigid perioperative timing constraints and manufacturing lead times inherent in autologous cell processing, the deployment of “off-the-shelf” allogeneic CAR-T cells (using T-cell receptor [TCR] gene knockouts to prevent graft-versus-host disease) offers a crucial logistical and feasibility advantage. Post-treatment surveillance should combine longitudinal liquid biopsies (CTC counts and ctDNA molecular kinetics) alongside conventional CA19-9 biochemical tracking and high-resolution cross-sectional imaging. To address biomarker assay standardization, primary efficacy endpoints should center on a composite metric of early molecular clearance–defined as CTC counts below the baseline limit of quantification (LOQ) and absolute plasma ctDNA negativity at day +30 and during early surveillance—alongside standard survival metrics including RFS and OS [70,76,77].

The clinical development strategy should begin with a first-in-human Phase I dose-escalation study evaluating safety and dose-limiting toxicities (DLTs) across separate cohorts of metastatic, borderline-resectable, and resectable PDAC patients to establish rigorous toxicity-monitoring guidelines. Subsequently, Phase II efficacy evaluations should use a randomized design with defined comparator arms: patients with resectable PDAC treated with the current standard of care (R0/R1 surgical resection followed by standard adjuvant chemotherapy) versus the standard of care combined with perioperative CEACAM6 CAR-T cell interception. Efficacy endpoints should focus primarily on RFS, as this is the most sensitive parameter for evaluating the mitigation of early systemic relapse risk.

### 3.11. The Correlation vs. Causation Conundrum

In closing this translational analysis, a critical scientific caveat must be explicitly emphasized: all contemporary data linking CTC metrics to PDAC outcomes are strictly prognostic and observational rather than interventional. Robust multicenter trials and meta-analyses have consistently shown that the pre-operative or intra-operative presence of CTCs is associated with significantly shorter disease-free and overall survival [94,95]. However, the clinical oncology community currently lacks direct evidence that the active, therapeutic elimination of these circulating cells improves patient survival or alters the natural history of PDAC. It remains biologically plausible that the presence of numerous CTCs is merely a passive epiphenomenon, reflecting an aggressively proliferating primary tumor that has already established widespread, undetectable microscopic niches. In this case, only the active elimination of CTCs and their micro-metastatic seeds by CAR-T cells would be effective. Conversely, clearing cells in transit in metastatic patients may not impact clinical output. Therefore, the CEACAM6 CAR-T framework proposed herein must not be interpreted as a verified therapeutic solution, but strictly as a translationally sound, highly targeted experimental hypothesis. Prospective, tightly regulated early-phase clinical trials are urgently required to bridge this fundamental gap, shifting the field from observational tracking to interventional verification, and definitively proving whether liquid-phase interception can yield true survival benefits.

## 4. Conclusions

Given the early systemic dissemination, the strong prognostic value of CTCs, and the growing feasibility of MRD-guided immunotherapy, targeting CTC in PDAC at MRD state is rational—particularly with CEACAM6 as a biologically relevant target and CAR-T as the effector. Hematology experience validates the MRD-interception principle; the next step is to prove that biomarker clearance translates into fewer relapses and longer survival in PDAC [7,66,70]. CTC-targeting in PDAC is biologically plausible, clinically measurable, and ready for prospective testing. CEACAM6-directed CAR-T therapy positioned as peri-operative MRD interception in CTC/ctDNA-positive patients seems a rational development path. Success will depend on standardized CTC assays, safety-engineered cell products, and rigorous endpoints that link MRD clearance with definitive clinical outcomes, namely fewer relapses and improved survival.

## Figures and Tables

**Table 1:** PDAC patient classification, treatment strategies, and CTC levels by resectability group.

Resectability Group *	Typical Treatment	CTC Level **	Overall Survival
Primary resectable	Surgery + adjuvant chemotherapy	Low (7–11 CTC/mL) [6]	high
Borderline resectable	Induction chemotherapy ± surgical resection ± Adjuvant chemotherapy	Middle (11 CTC/mL) [6]	middle
Locally advanced, unresectable	Induction chemotherapy ± surgical resection or systemic treatment with palliative intent	High (20 CTC/mL) [6]	low
Metastatic	Systemic treatment with palliative intent and/or best supportive care	High (20 CTC/mL) [6]	low

* Based on NCCN Guidelines for Pancreatic Cancer v2.2025 [18,19]: ** Based on CLUSTER study results for total CTC count [6].

**Table 2:** Evaluation of tumor-associated antigens in pancreatic ductal adenocarcinoma (PDAC): expression prevalence, biological roles, and clinical therapeutic landscapes.

Antigen	Evidence in PDAC/Biology	Clinical Notes
CEACAM6	Very frequent in PDAC (approx. 80–90%); adhesion/FAK–Src; anoikis resistance [52]	Primary CAR-T target; verify on CTCs; watch off tumor-T target;
TROP2	Overexpression in 30–68% of patients [53]	Sacituzumab govitecan (Trodelvy^®^), TROP2-directed ADC in Phase II
Nectin4	Overexpression in approx. 54% of patients [54,55]	Target for Enfortumab vedotin (Padcev^®^)–ADC in Phase II
Mesothelin (MSLN)	Overexpressed in approx. 80–90%; metastasis/immune contexture [56]	Limited intratumor efficacy signals [50] Anetumab ravtansine (Bayer) in mesothelioma, gynecologic, and other cancers; strong preclinical activity but mixed phase II results; the development of anetumab ravtansine was stopped [trial: NCT03926143]
MUC1/MUC1-C	Overexpressed in approx. 80–95% aberrant glycosylation; active ADC/immune pipeline-metastatic [57]	Contingency/combination; glycoformaware binder-aware binder
Claudins (CLDN4/18.2)	Tight junction markers enriched in PDAC; emerging translational data; approx. 85–95% expression [58,59]	Registered in the treatment of gastric cancer with Claudin expression
EGFR	Common, associated with dedifferentiation and poorer survival, overexpression approx. 60–65% [60]	Broad expression → affinity tune; strict CTC gating-tune
PSCA	Reported in PDAC (approx. 60%); signals in myeloid/NKT cell therapies [61]	Explore platforms beyond CART; verify surface density-T

## Data Availability

No new data were created or analyzed in this study.

## Abbreviations

PDAC	Pancreatic ductal adenocarcinoma
CTC	Circulating tumor cell
ctDNA	Circulating tumor DNA
Ca 19.9	Carbohydrate Antigen 19.9
MRD	Minimal residual disease
EUS	Endoscopic ultrasound
PoVB	Portal venous blood
PB	Peripheral blood
CAR-T	Chimeric antigen receptor T cell
CAR-NK	Chimeric antigen receptor natural killer cell
TIL	Tumor-infiltrating lymphocyte
TCR-T	T-cell receptor–engineered T cell
MSI-H/dMMR	Microsatellite instability–high/deficient mismatch repair
RFS	Relapse-free survival
DFS	Disease-free survival
OS	Overall survival
PFS	Progression-free survival
LOQ	Limit of quantitation
LOD	Limit of detection
